# Three-Dimensional Dental Analysis for Sex Estimation in the Italian Population: A Pilot Study Based on a Geometric Morphometric and Artificial Neural Network Approach

**DOI:** 10.3390/healthcare10010009

**Published:** 2021-12-22

**Authors:** Giorgio Oliva, Vilma Pinchi, Ilenia Bianchi, Martina Focardi, Corrado Paganelli, Rinaldo Zotti, Domenico Dalessandri

**Affiliations:** 1 School of Dentistry, Department of Medical and Surgical Specialties, Radiological Sciences, and Public Health, University of Brescia, Piazzale Spedali Civili 1, 25123 Brescia, Italy; giorgio.oliva1@gmail.com (G.O.); corrado.paganelli@unibs.it (C.P.); r.zotti@unibs.it (R.Z.); domenico.dalessandri@unibs.it (D.D.); 2 Section of Forensic Medical Sciences, Department of Health Sciences, University of Florence, Largo Brambilla 3, 50134 Florence, Italy; vilma.pinchi@unifi.it (V.P.); martinafocardi@gmail.com (M.F.)

**Keywords:** geometric morphometric analysis, artificial neural network, multilayer perceptron, sex estimation, 3D dental images, forensic odontology, dental sexual dimorphism

## Abstract

Dental dimorphism can be used for discriminating sex in forensic contexts. Geometric morphometric analysis (GMA) allows the evaluation of the shape and size, separately, of uneven 3D objects. This study presents experiments using a novel combination of GMA and an artificial neural network (ANN) for sex classification, applied to premolars of Caucasian Italian adults (50 females and 50 males). General Procrustes superimposition (GPS) and the partial least square (PLS) method were performed, respectively, to study the shape variance between sexes and to eliminate landmark variations. The “set-aside” approach was used to assess the accuracy of the proposed neural networks. As the main findings of the pilot study, the proposed method applied to the first upper premolar correctly classified 90% of females and 73% of males of the test sample. The accuracy was 0.84 and 0.80 for the training and test samples, respectively. The sexual dimorphism resulting from GMA was low, although statistically significant. GMA combined with the ANN demonstrated better sex classification ability than previous odontometric or dental morphometric methods. Future research could overcome some limitations by considering a larger sample of subjects and other kinds of teeth and experimenting with the use of computer vision for automatic landmark positioning.

## 1. Introduction

The sexual dimorphism of human dentition can be useful in a variety of forensic settings, such as mass disasters, the identification of bodies, and the classification of skull fragments [1]. Several methods, such as genetic and skeletal morphology analysis, can be effectively used for sex estimation, but in some cases, the available evidence is insufficient for conclusive sex identification. For instance, sexual dimorphism can be studied in the dentition of young children, whose skeletons have not yet shown differences that become manifest during puberty [2]. Sexual dimorphism of the teeth is determined by genetics [3], masticatory function [4], and hormones [5]. However, sexual variations in dental size and shape tend to be population-specific. Some studies have reported a higher sexual dimorphism for maxillary teeth [6,7], especially premolars [8]. Previous studies have demonstrated the reliability of odontometric methods based on linear and angular measurements carried out on dental casts or 3D casts, obtained via scanning. Especially in forensic contexts, an intraoral scanner is seldom available during the post-mortem examination of the body; thus, analysis and measurements are frequently performed later on casts. In this sense, methods tested using dental casts, rather than real dentition, produce results that are actually more implementable in the current practice of forensic odontologists, and the evidence gained is not biased with the use of methods originally tested in vivo.

Methods based on three-dimensional analysis have demonstrated a higher capability in detecting variation of shape compared to two-dimensional analyses based on linear and angular measurements [9,10,11,12,13,14,15,16,17,18,19]. Nevertheless, these methods require one to consider several measures simultaneously and to produce an overall interpretation of morphological features based on separate shape registrations. Alternatively, some novel approaches have turned to more complex measurement approaches, such as geometric morphometric analysis (GMA) [20,21], which allows the evaluation of shape and size separately.

GMA applied to teeth does not select some parts of the tooth arbitrarily for the analysis and does not require separate computations. The entire occlusal surface of the crown is indeed considered, all the measurements and analysis are performed at the same time, and the results regarding similarities or variations in the compared dental crowns can be easily visualized [22]. GMA is based on both fixed landmarks and sliding semi-landmarks, and it therefore represents an optimal method to study three-dimensional surfaces [23]. Sliding semi-landmarks enables the analysis of uneven three-dimensional surfaces, where placing fixed landmarks could be difficult and biased.

Polychronis et al. [21] first applied GMA for exploring the sexual dimorphism in molars and only a variation in size, and not in the crown shape, emerged between sexes. Sorenti et al. [24] examined 2D mesial planes of MicroCT scans of mandibular molars through the logistic analysis of eight variables. The relative dentine area in molars was found to be the best predictor of sex, with on overall accuracy of 74.36%. The previous literature has mainly considered the size and crown features of molars for estimating ancestry or sex of individuals, even though they normally show higher rates of tooth wear, which can bias morphometric studies of tooth crowns [25]. 

Few studies have considered bicuspids. Among these is a study conducted by Yong et al. [18], who used landmarks and sliding semi-landmarks on maxillary and mandibular premolars for the analysis of sexual dimorphism and ancestry variations among different population groups. They used GMA and two-way Procrustes ANOVA (Analysis of Variance) to test group differences for ancestry and sex, finding upper bicuspids to be more accurate than mandibular ones in correctly classifying individuals into their ancestral groups. GMA or other methods which enable separate measurements of size and shape have been found to be promising, but they require relevant computational efforts for analyzing metric measures or geometric approximations of tooth shape. In fact, geometric morphometric data result from factor analysis, which produces vectors that are not easy to interpret as standard linear measures. Moreover, graphical visualization results are quite useful in interpreting the similarities of the compared tooth crowns, but produce no quantitative values that are usable for the classification of subjects. These limitations could be overcome with the use of artificial neural networks (ANNs), which provide learning algorithms based on input data.

The use of ANNs combined with GMA has been compared with classical methods of classification (linear or quadratic discriminate analysis) by Soda and et al. [26]. They demonstrated that ANNs yielded the most stable accuracy among the analyzed groups. The most commonly used ANN is the multilayer perceptron, which can easily interpret complex variables based on GMA to build an efficient classification algorithm. Therefore, ANNs could have significant potential in sex classification if measurement data obtained from GMA can be applied.

Therefore, in this study we aimed to test a new approach based on the combination of GMA and ANN applied to human premolars for detecting sexual dimorphism and then for classifying subjects by sex.

## 2. Materials and Methods

A total of 100 dental casts from Caucasian Italian adults (50 females and 50 males) who underwent dental impressions for clinical reasons were analyzed (mean age: 40 ± 7 years). According to the local Ethics Committee recommendations, patients gave their informed consent for the anonymous use of their dental casts for the study. The inclusion criteria were: patients without missing teeth, dental decay, pathologic anomalies of enamel and enamel/dentin, significant wear or a remarkable medical history.

The exclusion criteria were: patients who did not meet one or more inclusion criteria and gross irregularity of the cast.

The first left upper premolar was analyzed in this study. The maxillary first premolar was selected because it demonstrated a lower rate of tooth wear than other teeth [25] and high sexual dimorphism [8]. Additionally, only one tooth was examined in order to exclude any possible source of variability, although Kranioti et al. [16] demonstrated that symmetry exists between measurements of the left and right maxillary and mandibular teeth.

The casts were digitized with an iCarestream Dental Cs 3600 intraoral scanner (Carestream Dental, Stuttgart, Germany). The scans and landmark digitations were performed by an orthodontist experienced in this kind of analysis. A complete automation of the neural network learning for 3D classification could not be performed here, due to the limited sample and the fact that this was a pilot study, mainly addressed at exploiting the advantages of a GM analysis combined with ANN. In fact, the automatic positioning of landmarks or semi-landmarks requires adequate learning based on larger amounts of data and samples [27,28].

**Figure 1:**
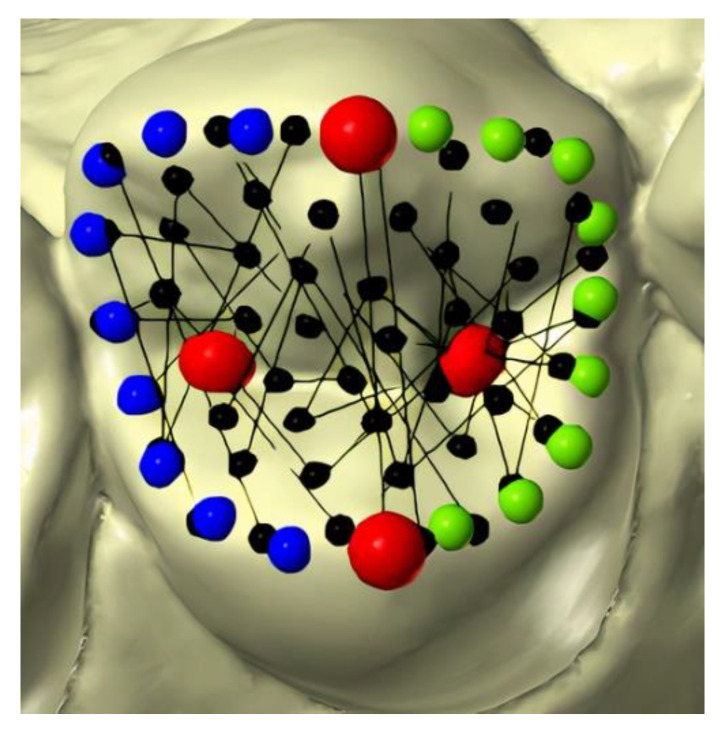
The distribution of landmarks (red points) and semi-landmarks (blue, green, and black points) on the tooth surface.

The occlusal morphology of premolars was studied by means of 3D geometric morphometrics. Landmark digitization was performed using Viewbox 4.0 software (dHAL software; Kifissia, Greece) and Yong’s scheme [18] was applied, since it was deemed suitable for morphometric analysis and the semi-landmark sliding method (Figure 1). In this paper, the authors used Yong’s benchmark protocol for the placement of landmarks and semi-landmarks [18]. Moreover, the model proposed here allows one to orient the 3D image and to perform a double check of the positions of reference points during digitation. In particular, fixed landmarks are first positioned according to the side view and then adjusted in the occlusal view.

In particular, the following landmarks were placed partly manually by the operator and partly automatically by the software:

1. Four fixed landmarks were manually placed on each tooth: the buccal and lingual cusp tips and the mesial and distal fossae. All the landmarks were initially projected from occlusal view and double checked by rotating the models (red points).
2. Nine semi-landmarks were placed manually to identify major ridges and to delimitate the occlusal circumference. To accomplish this, these two curves were drawn over the mesial and distal ridges, respectively, connecting the buccal and lingual cusp tips. The software automatically placed equally spaced semi-landmarks along each curve (blue and green points).
3. Fifty surface semi-landmarks were manually added on the occlusal circumference. This configuration was transposed to all the specimens using thin plate spline transformation (black points).
4. The curve and surface semi-landmarks were slid to minimize the bending energy between each premolar configuration and the reference specimen. Then, the semi-landmarks were automatically re-projected six times on their curves or surfaces [29].

To study premolar shapes, a general Procrustes superimposition (GPS) was performed. This procedure extracts shape information by eliminating the variations of landmark configurations that occur due to scaling (size differences), position, and orientation. Size differences are defined by centroid size. This is the root sum squared distance of all the landmarks and semi landmarks from their centroid [29]. Centroid size is a complex measurement that was proven to be significant in sexual dimorphism analysis by Polychronis et al. [21].

The significant differences in centroid size between male and female subjects were statistically tested with a *t*-test.

The intra- and interrater agreement was measured based on the digitation of 30 randomly selected casts, carried out 30 days after the first measurements and provided respectively by the same operator and a second orthodontist.

The Procrustes superimposition was then performed and the distance between landmark configurations in the shape space was used to measure the shape variance in the whole sample, as described by Klingemberg et al. [30].

In particular, partial least square (PLS) analysis was performed for all full landmark configurations. This method explores patterns of covariation between two blocks of variables and can be used to analyze the relationships between shape and other variables. PLS applied to Procrustes superimposition detects shape information by eliminating the landmarks’ variations due to size, orientation, and position. The results of these analyses are directions (call axes) that maximize covariation between variables. The significance of the covariation is tested using the permutation test (10,000 rounds).

The “set-aside” approach was used to assess the accuracy of the proposed neural networks [20,31] by diving the sample into two subgroups: the training sample (75 casts) used to build the ANN and the test sample (25 casts), on which the obtained ANN was applied in order to measure its performance in predicting sex. For sex classification, two variables were used as input variables (predictors) in the ANN: the first axis of the PLS analysis and the centroid size.

All neurons had sigmoidal activation and the optimization was perform using stochastic gradient descent. The appropriate number of hidden neurons was determined using the 10-K cross validation method to maximize the prediction accuracy. The threshold for group allocation was determined using ROC analysis.

The performance of the obtained ANN in predicting sex was measured in the test sample by assessing sensitivity, specificity, positive predictive value (PPV) and negative predictive value (NPV) for each sex, and an overall accuracy value.

All analyses were carried out using the R packages geomorph (https://cran.r-project.org/package=geomorph (Accessed on 20 July 2021)), neuralnet (https://github.com/bips-hb/neuralnet (Accessed on 20 July 2021)), and ROCR (http://rocr.bioinf.mpi-sb.mpg.de (Accessed on 20 July 2021)).

## 3. Results

The intra- and interrater agreement was evaluated based on the Procrustes distance obtained from the first and the second digitations carried out on 30 randomly selected casts, and the results were, respectively, 0.95% (intra-rater) and 0.98% (inter-raters). Only 1% of the total shape variance was found to be due to measurement errors. This percentage is similar to that found by Yong et al. and is within acceptable limits [20].

**Figure 2:**
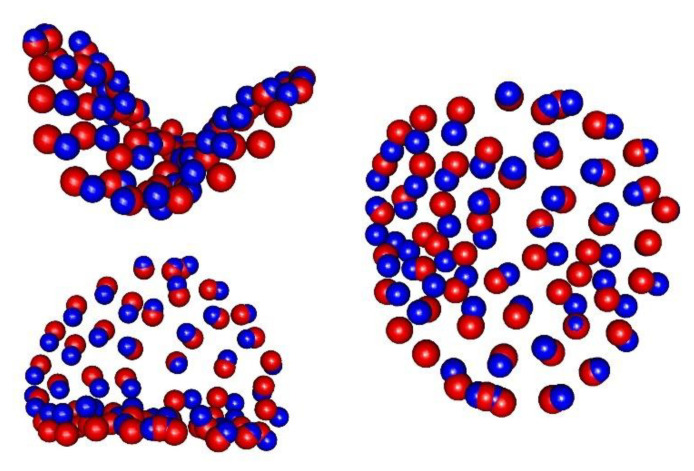
The image shows the differences between male and female premolar shapes after Procrustes superimposition and PLS analysis. Red landmarks indicate the baseline (female shape), and blue landmarks indicate an increase in the singular vector of PLS (male shape).

Figure 2 shows the differences in premolar shape between the male and female casts after GPS and PLS, performed on the Procrustes values obtained for the whole sample (100 casts). Female mean shape is taken as the baseline (red points). The first axis of the PLS displaces red points in the direction that maximizes the difference between females and males (blue points). Male subjects showed larger crowns and well-defined ridges, but such variations are not usable to correctly classify subjects by sex.

The most important differences were found at the buccal cusp, along the mesial and distal ridges, and in the mesial and distal fossae. The correlation between the first axis and sex was fund to be 0.73, and the *p*-value calculated with a permutation test indicated significance at less than 0.05. The difference in centroid size according to sex was statistically significant, with a *p*-value less than 0.05. According to this, the centroid size and the first axis can be used as input parameters for the sex classification algorithm.

**Figure 3:**
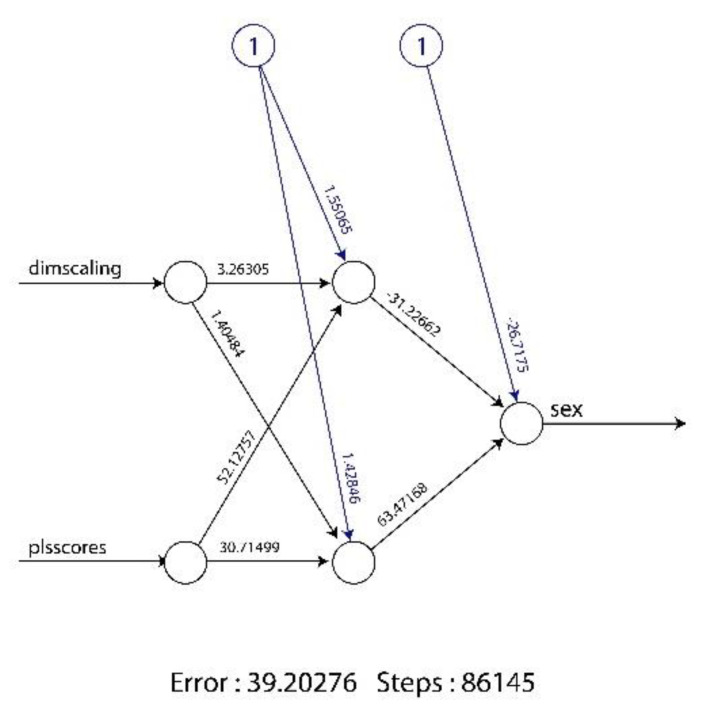
The artificial neural network created with the training sample.

For the realization of the ANN, the number of hidden layers was two and they were chosen using the 10-K cross-method. Two neurons were included in the first layer and one neuron was included in the second layer (Figure 3). “Dimscaling” represents the centroid size and “plsscores” denotes the results of the PLS analysis of the Procrustes superimposition. The numbers on the lines indicate the weight associated with each vector. Circles represent nodes of the ANN, distributed on three layers.

**Figure 4:**
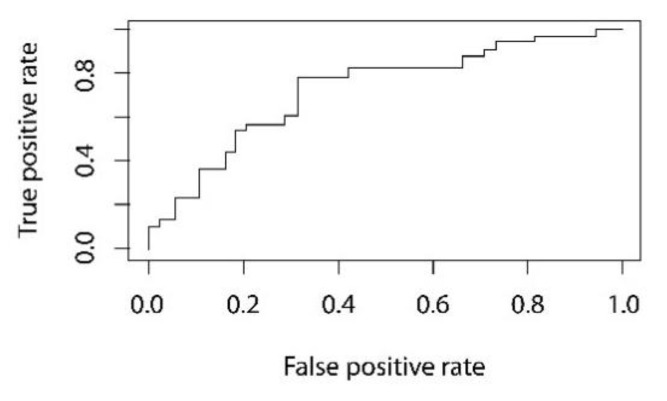
ROC analysis of the discrimination accuracy of the ANN. A threshold of 0.65 was used to optimize true-positive and false-positive rates.

The discrimination threshold between males and females was set at 0.65 via ROC analysis (Figure 4), since this value optimizes true-positive and false-positive rates.

The overall accuracy in classifying subjects by sex was found to be, respectively, 84% for the training sample and 80% for the test sample. The ANN yielded different sensitivity and specificity values for females and males (Table 1) in the test sample. The post-test probabilities (PPV and NPV) revealed that females showed a higher probability of being correctly positively classified as females, whereas males showed a higher probability of not being misclassified as females.

## 4. Discussion

Odontometric methods was proven to be a reliable tool in estimating the sex of individuals in forensic contexts and several studies have reported variations in the size or shape of teeth between male and female subjects in different populations. Metric analysis greatly prevailed in methods addressed at detecting shape variations between sexes, whereas very few combined analyses of size and shape have been carried out, and even less have used ANNs to perform the computational efforts that such combinations require. This study tested whether a method based on geometric morphometric analysis (GMA) combined with an ANN for studying the sexual dimorphism of teeth could overcome some of the limitations discussed in the previous literature, while yielding an accurate identification of sex.

According to some prior studies, this study was based on upper premolars, which are less affected by attrition compared to other teeth and have been found to be endowed with sexual dimorphism [8,25]. Moreover, dental casts were preferred for measurements, instead of measures or scans taken in vivo. In forensic daily practice, an intra-oral scanner is very rarely available in morgue facilities and measures and analysis of dentition are often performed later on casts and not during oral autopsies. Geometric morphometric analysis, performed with the use of ANN on dental casts, was first demonstrated to be a highly reproducible method, since the intrarater and interrater agreement indexes were found to be 0.95% and 0.98%, respectively.

The first analysis, carried out on the whole sample, revealed a certain sexual dimorphism between the upper first premolars of males and females, showing the males to have larger crowns and more well-defined ridges compared to females. In agreement with other studies [20,21] these differences, although statistically significant, were found to be insufficient for discriminating the sex of a subject. Thus, the centroid size was added as a parameter in the ANN.

In contrast with most previous studies, the performance of the proposed approach was measured on a test sample, and not evaluated on the overall sample used to build the method.

The first relevant observations were related to the fact that the most accurate values obtained in discriminating sex were found in female subjects, rather than in males. These findings are consistent with several previous reports [7,8,10,13,19]. Mujib et al. [13] took into consideration diagonal crown measurements of upper canines and molars and found the method to be accurate in 69% of males and 73% of females. Işcan et al. [10] analyzed linear and angular measurements of all maxillary and mandibular teeth and found an accuracy of 80% in females and of 72% in males. Similar results were reported by Acharya [6] for dentition in a Nepalese population. On the contrary, Kranioti et al. [16] and Tabasum et al. [17] analyzed upper molars to discriminate between sexes and found higher accuracy for males (84–87%) compared to females (53–70%). Slightly higher accuracy in Indian males than in female subjects was also reported by Yadav et al. [14] and Prabhu and Acharya [12] in an Indian population. These reports, globally considered, seem to indicate that sex-related dental traits tend to be more pronounced in females or in males in different populations.

Very few previous studies have considered the accuracy of methods based on one single tooth and have reported poor capability in discriminating the sex of the subject. Singh et al. found accuracy values in classifying the sex of individuals that ranged from 40% to 60% for premolars and slightly higher values (58–66%) for other kind of teeth studied separately [19].

GMA has demonstrated to be very effective for the separate analysis of dental shape and size both for assessing sexual dimorphism and for classifying subjects by sex [18]. Since GMA can reproduce variable objects and yield highly representative data [26], it was applied for handling complex three-dimensional dental images obtained from the scanned upper premolars of the sample. However, the shape representations obtained through the Procrustes superimposition of the premolars of males and female subjects were found to be unsuitable per se for classifying subjects by sex.

A partial-least-square (PLS) analysis was then performed to study the variance of the Procrustes superimposition, and an ANN was applied to cope with the required computations and analysis. In fact, the previous literature has demonstrated that GMA combined with an ANN can be used to obtain better prediction for species classifications [26], especially if dimensionality reduction is performed [31]. Therefore, we used GMA to obtain shape data from the sample, a PLS analysis to reduce the dimensionality of the data, and a multilayer perceptron type of ANN in order to obtain a better classification of the sample.

The discriminating capabilities of the studied ANN for the analysis of a single tooth allowed very high rates of overall accuracy for both the training sample and the test sample, with values of 0.84 and 0.80, respectively. The proposed method yielded very high rates of sensitivity for males (92%) and specificity (92%) for females. The ROC analysis yielded a generally very good area under the curve (AUC), with 0.81 as the value used to optimize the specificity and sensitivity.

The post-test probability values (PPV and NPV) indicate the probability of a subject to be correctly attributed to the proper sex group, and females were found to have a high probability of being correctly positively classified as females, whereas males showed the same probability of being not misclassified as females. 

The accuracy values were found to be 90% for females and 73% for males.

These preliminary results appear quite promising in comparison with previous studies on the use of premolars or different teeth (canines and molars) for classifying individuals by sex in different populations [9,10,11,12,13,14,15,16,17,18,19,20,21].

In fact, similar very good performances were reported by previous studies only when all or several teeth were included in the analysis. Acharya et al. [6] reported accuracy values of 90% for males and 92.5% for females, but only when mesiodistal and buccolingual measures of all maxillary and mandibular teeth were included. The accuracy decreased progressively and considerably when either mandibular or maxillary teeth, groups of teeth, or single teeth were considered [6,10,12,17,19]. The methods which require measurements of all teeth suffer from evident limitations, as they are not applicable in case of missing teeth and are much more time consuming compared to methods based on few or one single tooth. Moreover, it should be noted that these studies offered limited or no validations of the used technique on a test sample. Thus, the real accuracy and the performance of such methods could be found to be lower, if applied in a sample that is different from that used to set the functions.

To our knowledge, only Yong [20] has studied premolars through the use of GMA and a Procrustes ANOVA analysis in order to develop a model for classifying Australians according to ancestry groups (European and Indigenous) and sex. The method yielded relevant accuracy rates for ancestry, but poor overall accuracy for the sex classification of individuals. The shape analysis of premolars showed accuracy values less than 70% of the time, whereas the centroid size was found not to be accurate in predicting the sex of individuals. The remarkably better results obtained in the present study could originate mainly from the application of ANNs that can use different kinds of predictors combined in a “nonlinear” prediction algorithm. Moreover, Yong’s study was mainly addressed at estimating ancestry and the considered sample of females and males for each ancestry group was quite limited.

Therefore, the tested combination of GMA and ANNs for the assessment of the sex of an individual based only on one tooth (upper premolar) emerged as an accurate and reliable method compared to previous techniques which require more computational efforts and the analysis of all or numerous teeth.

Nevertheless, future research should involve the analysis of other teeth, such as molars or canines, in order to explore possible improvements of sex classification by combining different kinds of teeth. Moreover, a small group of teeth considered in the analysis has been deemed desirable for managing those cases in which the target tooth is missing, without pursuing methods based on measures of all teeth, which carry the risk of being even less applicable in cases where teeth are missing.

The main limitations of this study were due to the small sample size considered in this pilot study and the consequent limits posed by GMA. Even though the sample size was sufficient to train two predictor neural networks, the training of a bigger and better neural network requires a larger sample. In this paper, PLS analysis was used to obtain a reduction of the data, because this yields fewer, but more significant predictors than principal component analysis (PCA). However, in future studies involving bigger samples, differences between PLS reduction and PCA reduction should be evaluated, considering that an unsupervised learning approach is often preferred [32] (pp. 115–116). Moreover the feasibility of the method relies on specific software capabilities for both the analysis of 3D-digital images and the realization of the neural network. Finally, an ANN needs to be trained under some imposed conditions (activation function, number of hidden layers, and others), and this often relies on a trial-and-error approach. Hence, future studies should consider the use of computer vision for automatic landmark placement.

Despite these limitations, the combination of GMA and the ANN demonstrated a higher prediction capability than previous methods based on linear or angular measures or the shape analysis of dental crowns for classifying subjects by sex.

## 5. Conclusions

In this pilot study, we assessed the application of GMA combined with ANNs for analyzing the upper first premolar sexual dimorphism and the method’s performance in classifying subjects by sex. The proposed approach was found to be endowed with very high repeatability. Despite the small size of the analyzed sample, GMA combined with ANNs demonstrated very good sex classification ability compared to previous odontometric or dental morphometric methods based on linear and angular analysis or simple linear discrimination analysis of multiple teeth. In this study, measurements of the first upper premolar were used to correctly classify 90% of females and 73% of males of the test sample after a neural network was specifically trained with predictors derived from the GMA set on the training sample. In general, the present findings contribute to increasing the scientific evidence that supports the implementation in the forensic field of methods based on GMA and ANNs, which were found to be less demanding from a computational point of view and less affected by limitations, compared to linear and angular or shape analysis.

Future research should consider larger samples suitable for analyzing more predictors and other kinds of teeth, in order to confirm the reliability of the results obtained here for a small, but actually sufficient, sample to study two dental variables in one single tooth. Further studies should investigate different types of deep learning approaches based on larger samples of subjects, other kinds of teeth, and more predictors, aiming towards the standardization of the positioning of landmarks and the automation of morphometric analyses, thereby minimizing the influence of operator subjectivity.

## Figures and Tables

**Table 1:** Sensitivity, specificity, positive predictive value, and negative predictive value stratified by sex. The accuracy values were 80% and the ROC analysis yielded an overall value of 0.81.

	Females	Males
**Sensitivity**	70%	92%
**Specificity**	92%	70%
**PPV**	90%	73%
**NPV**	73%	90%
**Total Auc of ROC analysis**	0.81	
